# Evaluation of the effect of modafinil on the pharmacokinetics of encorafenib and binimetinib in patients with BRAF V600-mutant advanced solid tumors

**DOI:** 10.1007/s00280-024-04676-2

**Published:** 2024-06-15

**Authors:** Joseph Piscitelli, Micaela B. Reddy, Lance Wollenberg, Laurence Del Frari, Jason Gong, Kyle Matschke, Jason H. Williams

**Affiliations:** 1 Pfizer Inc, 10555 Science Center Dr, San Diego, CA 92121 USA; 2 Pfizer Inc, Boulder, CO USA; 3 Pierre Fabre Medicament, Toulouse, France; 4 Pfizer Inc, New York, NY USA; 5 Pfizer Inc, Collegeville, PA USA

**Keywords:** Oncology, Drug-drug interaction, Modafinil

## Abstract

**Background:**

A clinical drug-drug interaction (DDI) study was designed to evaluate the effect of multiple doses of modafinil, a moderate CYP3A4 inducer at a 400 mg QD dose, on the multiple oral dose pharmacokinetics (PK) of encorafenib and its metabolite, LHY746 and binimetinib and its metabolite, AR00426032.

**Methods:**

This study was conducted in patients with BRAF V600-mutant advanced solid tumors. Treatment of 400 mg QD modafinil was given on Day 15 through Day 21. Encorafenib 450 mg QD and binimetinib 45 mg BID were administered starting on Day 1. PK sampling was conducted from 0 to 8 h on Day 14 and Day 21. Exposure parameters were calculated for each patient by noncompartmental analysis and geometric least-squares mean ratio. Corresponding 90% confidence intervals were calculated to estimate the magnitude of effects.

**Results:**

Among 11 PK evaluable patients, encorafenib C_max_ and AUC_last_ were decreased in presence of steady-state modafinil by 20.2% and 23.8%, respectively. LHY746 exposures were not substantially changed in the presence of steady-state modafinil.

**Conclusion:**

The results from this clinical study indicate modafinil 400 mg QD had a weak effect on encorafenib PK. Based on these results, encorafenib can be coadministered with a moderate CYP3A4 inducer without dosing adjustment.

**Clinical trial registration:**

ClinicalTrials.gov NCT03864042, registered 6 March 2019.

## Introduction

Encorafenib is a potent and selective, oral, adenosine triphosphate (ATP)-competitive small-molecule inhibitor of BRAF V600-mutant kinase. The recommended dose of encorafenib is 450 mg once daily in combination with binimetinib (45 mg twice daily) for the treatment of metastatic melanoma as well as metastatic non-small cell lung cancer and 300 mg once daily in combination with cetuximab (400 mg/m^2^ initial dose, followed by 250 mg/m^2^ weekly) for the treatment of metastatic colorectal cancer. CYP3A4 was the major enzyme contributing to ∼ 83% of total oxidative clearance of encorafenib in human liver microsomes and encorafenib is therefore susceptible to DDIs when coadministered with inhibitors or inducers of this enzyme [1]. It has been demonstrated in a clinical DDI study that coadministration of strong CYP3A inhibitor posaconazole and moderate CYP3A4 inhibitor diltiazem caused 3-fold and 2-fold increases in encorafenib exposures, respectively [2]. These findings led to the recommendation that coadministration of encorafenib with strong or moderate CYP3A4 inhibitors should be avoided, but if coadministered, to monitor patients closely for signs and symptoms of increased exposure and consider reducing the dose of encorafenib accordingly [1].

Recommendations for inducers based on in vitro studies were to avoid coadministration of encorafenib with strong or moderate CYP3A inducers. Prediction of the effect of induction in vivo is complicated by the finding that encorafenib exhibits auto-induction of CYP3A4, resulting in approximately 50-percent lower exposure at steady-state compared to following a single dose. This phenomenon was first observed clinically in the encorafenib first in human study in which this trend was observed at every dose level of encorafenib [3]. Trough plasma concentrations collected at the end of the first cycle in this same study did not show an appreciable difference in PK between 15 and 28 days after encorafenib initial dosing, suggesting that steady-state conditions had been reached by C1D15 [3].

A study to evaluate the effect of a perpetrator drug on steady state encorafenib concentrations must be conducted in patients rather than in healthy volunteers due to the risk of secondary skin neoplasms with selective BRAF inhibitors upon repeat dosing [4]. Evaluation of strong inducer’s effect on the PK of encorafenib was considered however the effect was expected to be significant (> 50% decrease in exposure) and this significant decrease could potentially lead to a loss of efficacy for the patients in this study [5]. Therefore, this phase 1 clinical DDI study (ClinicalTrials.gov ID: NCT03864042) was designed to evaluate the effect of multiple doses of modafinil, a moderate CYP3A4 inducer at a 400 mg QD dose, on the multiple oral dose PK of encorafenib. The recommended dose of encorafenib, 450 mg administered once daily (QD) in combination with binimetinib, 45 mg administered twice daily (BID), as indicated for patients with BRAF V600 metastatic melanoma, was used in this study.

While binimetinib has no known clinically relevant DDIs, concentration measurements were included in the study for binimetinib and its metabolite AR00426032. UGT1A1-mediated metabolism is thought to be the primary mechanism of elimination for binimetinib. Although the mechanism for CYP3A induction may also lead to induction of UGT1A1, no clinically significant differences in PK were observed when coadministered with encorafenib [6–8].

In addition, evaluation of LHY746, the primary yet inactive metabolite of encorafenib formed by CYP3A4-mediated metabolism, was included to add insight into the induction. The metabolism of LHY746 is mainly through CYP2C19 with some contribution of CYP2C9 and CYP3A4 (data on file).

## Methods

### Study population

This study was conducted in compliance with the ethical principles originating in or derived from the Declaration of Helsinki and in compliance with all ICH GCP Guidelines. In addition, all local regulatory requirements were followed, in particular, those affording greater protection to the safety of trial participants.

Males or females age were eligible for enrollment if they were ≥ 18 years of age, had histologically confirmed diagnosis of locally advanced, unresectable or metastatic cutaneous melanoma American Joint Committee on Cancer Stage IIIB, IIIC or IV; or other BRAF V600-mutant advanced solid tumors, with evidence of measurable or non-measurable lesions as detected by radiological or photographic methods according to guidelines based on Response Evaluation Criteria in Solid Tumors v1.1, had Eastern Cooperative Oncology Group (ECOG) performance status of 0 or 1 and adequate bone marrow, hepatic and renal function.

Key exclusion criteria included participants with symptomatic brain metastasis, history of reaction to any of the study medications, symptomatic or untreated leptomeningeal disease, history or current evidence of retinal vein occlusion (RVO) or current risk factors for RVO (e.g., uncontrolled glaucoma or ocular hypertension, history of hyperviscosity or hypercoagulability syndromes), clinically significant cardiac disease, impaired hepatic function as defined by Child-Pugh class B or C, impaired gastrointestinal function or disease which might have significantly altered the absorption of study drugs (e.g., ulcerative diseases, uncontrolled nausea, vomiting, diarrhea, malabsorption syndrome, small bowel resection), known hyper-coagulability risks other than malignancy (e.g., Factor V Leiden syndrome), thromboembolic event except catheter-related venous thrombosis ≤ 12 weeks prior to starting study treatment, discontinuation of prior BRAF and/or MEK inhibitor treatment due to left ventricular dysfunction, pneumonitis/interstitial lung disease, or RVO.

Participants were also excluded if they had use within 2 weeks prior to the start of encorafenib/binimetinib treatment on Day 1 and through DDI phase (Day 28), of any herbal medications/supplements or any medications or foods that are substrates, inhibitors or inducers of CYP3A4/5, consumption of grapefruit, pomegranates, star fruits, Seville oranges or products containing the juice. Due to the use of modafinil in this study, participants were also excluded if they had a history of psychosis, depression, mania, angioedema, mitral valve prolapse, or left ventricular hypertrophy.

### Study design

**Fig. 1:**
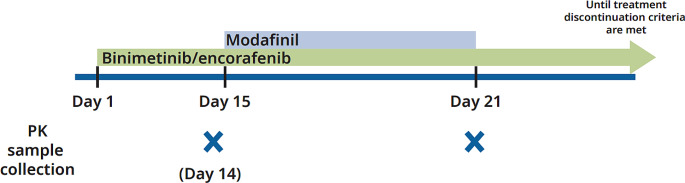
Study design Abbreviations: PK = pharmacokinetics

A schematic for the study design is presented in Fig. 1. Encorafenib 450 mg QD and binimetinib 45 mg BID were administered starting on Day 1. Patients then received continuous treatment of 400 mg QD modafinil on Day 15 through Day 21. Blood samples for measurement of plasma concentrations of encorafenib (and its metabolite, LHY746) and binimetinib (and its active metabolite, AR00426032), were collected at 0, 1, 2, 3, 4, 6 and 8 h post dose on Day 14 and Day 21.

### Rationale for modafinil dose

Modafinil was administered at 400 mg for 7 days to ensure sufficient time for CYP3A4 induction, while minimizing the time for which patients may have reduced exposures to encorafenib [9, 10].​ While modafinil is considered a weak inducer at 200 mg/day, at a higher dosage (400 mg/day) modafinil is considered a moderate inducer of CYP3A [11]. Furthermore, the effect of modafinil (200 mg for 7 days followed by 400 mg for 21 days) on CYP3A4 substrate triazolam PK was moderate, with a 59% decrease on mean AUC_inf_ [10]. Lastly, modafinil has been shown to be well tolerated with headache as the only observed AE in > 15% of patients [12].

### Bioanalytical procedures and PK analysis

Human PK plasma samples were analyzed for quantitation of all PK analytes at PPD (Middleton, Wisconsin, US) using validated, sensitive, and specific HPLC/MS/MS methods in compliance with laboratory SOPs.

PK parameters including, but not limited to, C_max_ and AUC_last_ were calculated for each participant using noncompartmental analysis (NCA) methods using the WinNonlin software package (Phoenix WinNonlin Professional, version 8.0 Pharsight Corporation, Mountain View, California) for PK analytes including encorafenib and its metabolite LHY746, and binimetinib and its metabolite AR00426032.

### Safety

Safety was assessed during the DDI phase (Day − 7, Day 1, Day 14, Day 15, Day 21, and Day 28) and in the post DDI phase every 3 to 4 weeks until discontinuation of study drug. Safety monitoring included SAEs, laboratory profiles (hematology, biochemistry, coagulation, cardiac/muscle enzymes, urinalysis), physical examination (including vital signs, ophthalmic and dermatological examinations), ECOG PS assessment, and cardiac profiles (ECG and MUGA or ECHO), concomitant medications and/or therapies. AEs were classified according to the Medical Dictionary for Regulatory Activities (MedDRA, http://www.meddra.org) classification system version 22.1 and graded according to the Common Terminology Criteria for Adverse Events (CTCAE) version 4.03.

### Statistical analysis

The PK data was analyzed after the first 6 patients were enrolled to look for an indication that the moderate inducer was having a significant effect on encorafenib PK. Since there was a ≥ 20% change in mean encorafenib AUC, an additional 6 patients were to be enrolled to more fully characterize the effect. Assuming an intrasubject variation of 36.4% for encorafenib AUC_tau_, there was an 80% probability with fixed sample sizes of 6 and 12 subjects that a treatment difference would be detected if the true effect size is 74% and 46%, respectively [13].

All patients who received at least one dose of any study drug were included in the Safety Set population. The Evaluable PK Set included all patients with sufficient concentration data to calculate at least one PK parameter for encorafenib and binimetinib on Days 14 and 21. Patients who discontinued, missed 3 or more consecutive doses of encorafenib prior to completion of the last PK sampling on Day 14, or required a dose reduction of encorafenib prior to completion of the last PK sampling on Day 14 were excluded from the Evaluable PK set. In addition, patients who missed any dose of study drugs on any of the PK days, or who vomited within 4 h after dosing on any of the PK days were excluded from Evaluable PK set.

An analysis of variance (ANOVA) was performed on the natural log (ln)-transformed Cmax and AUC_last_ of encorafenib, LHY746, binimetinib, and AR00426032. The least squares means (LSM) geometric mean ratio (GMR) and associated 90% confidence interval (CI) for each PK parameter were calculated using the exponentiation of the difference between treatment LSM from the analyses on the ln-transformed parameters and expressed as a percentage of Day 21 relative to Day 14.

## Results

### Study participants

A total of 15 participants were enrolled into this sub-study. All these patients received study treatment and completed the DDI phase; however, only 11 participants could be included in the Evaluable PK set. One participant was excluded due to missing greater than 3 consecutive doses of encorafenib before Day 14, one participant was excluded due to a modafinil prescription error, one participant was excluded due to a missed modafinil dose on day 21, and one participant was excluded due to aberrant (low) PK exposure of encorafenib on Day 14.

The participants had a mean age of 58.9 years (range 41–80 years), mean body mass index of 26.0 kg/m^2^(range 18.2–33.6), with the number of males and females evenly split (Table 1). White, non-smoker and ECOG status of 0 were most frequent of their respective categories. All 15 participants were stage IV and had BRAF-mutant advanced solid tumors. The most frequently reported primary cancer was melanoma and others were colorectal, pancreatic, breast, and ovarian cancers.

**Table 1:** Demographics and Baseline Characteristics

Trait	Category/Statistic	Value *N* = 15
Age (years, at screening)	*n*	15
	Mean	58.9
	SD	9.98
	Median	58.0
	Minimum	41
	Maximum	80
Sex, *n* (%)	Female	7 (46.7)
	Male	8 (53.3)
Race, *n* (%)	White	14 (93.3)
	Asian	1 (6.7)
Ethnicity, *n* (%)	Hispanic or Latino	0
	Not Hispanic or Latino	15 (100)
Height (cm, at screening)	n	15
	Mean	169.8
	SD	9.22
	Median	169.93
	Minimum	156.0
	Maximum	189.0
Weight (kg, at screening)	n	15
	Mean	75.1
	SD	15.7
	Median	73.2
	Minimum	52.2
	Maximum	97.1
BMI (kg/m^2^, at screening)^a^	n	15
	Mean	26.0
	SD	4.92
	Median	26.0
	Minimum	18.2
	Maximum	33.6
ECOG performance status^b^, *n* (%)	0	8 (53.3)
	1	7 (46.7)
Smoking Status, *n* (%)	Non-smoker	14 (93.3)
	Occasional Smoker	0
	Smoker	1 (6.7)
Tumor Type^c^	Melanoma	5 (33.3)
	Colorectal Cancer	3 (20.0)
	Pancreatic Cancer	3 (20.0)
	Tumor of Unknown Origin	2 (13.3)
	Breast Cancer	1 (6.7)
	Ovarian Cancer	1 (6.7)

^a^BMI[kg/m²] = weight[kg]/height[m]²

^b^ECOG performance status: 0 = Fully active, able to carry on all pre-disease performance without restriction; 1 = Restricted in physically strenuous activity but ambulatory and able to carry out work of a light or sedentary nature

^c^All patients were classified as Stage IV cancer at the study entry

### Pharmacokinetics and drug-drug interactions

#### Encorafenib and LHY746

**Fig. 2:**
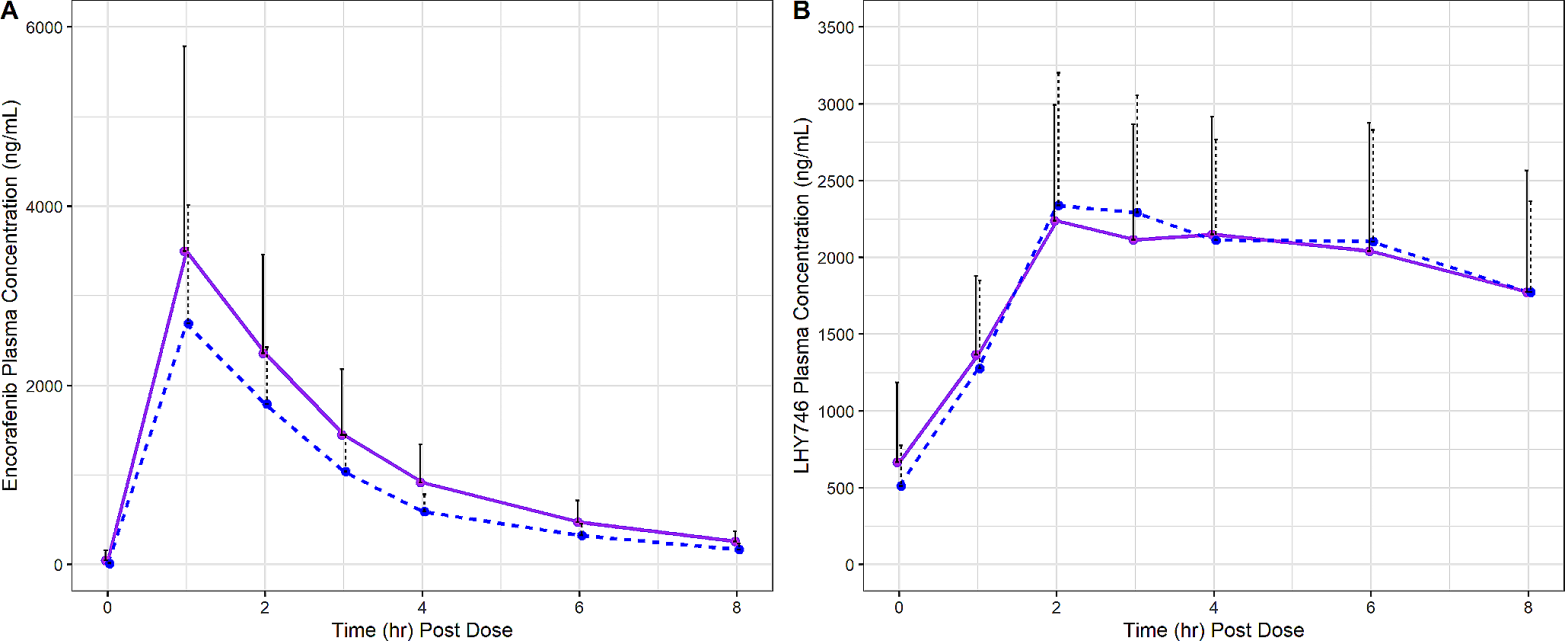
Mean (+ SD) Plasma Concentration-Time Profiles for Encorafenib and LHY746 by Study Day. The solid lines represent the data on Day 14 after encorafenib 450 mg QD and binimetinib 45 mg BID coadministration and the dotted lines represent the data on Day 21 after modafinil coadministration, 400 mg QD, beginning on Day 15 and extending through Day 21. The solid dots represent the mean analyte concentration at the time point specified. The black lines represent the upper standard deviation of the analyte concentration at each time point. Figure 2A presents encorafenib concentrations and Fig. 2B presents LHY746 concentrations

**Fig. 3:**
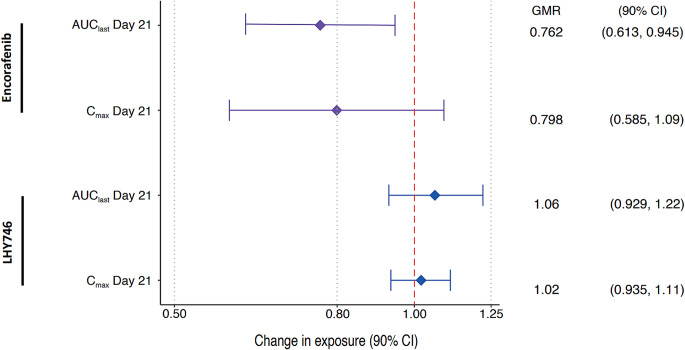
Effect of Steady-State Modafinil on Steady-state Encorafenib and LHY746 PK Parameters. Participants were administered 450 mg QD encorafenib and 45 mg BID binimetinib from Day 1 to the end of the study and 400 mg QD modafinil was coadministered from Day 15–21. AUC_last_, area under the curve from 0 to the last measurable point; CI, confidence interval; C_max_, maximum observed concentration; GMR, geometric mean ratio

Following repeated daily oral administrations of 450 mg encorafenib and 45 mg binimetinib BID on Days 1 through 21, C_max_ of encorafenib was attained (T_max_) at 1 h postdose on both Days 14 and 21 (Fig. 2; Table 2). Thereafter, plasma concentrations of encorafenib declined with geometric mean t_1/2_ values of 3.48 and 3.57 h on Days 14 and 21, respectively. Encorafenib exposures on Day 14 were consistent with historical studies [3]. The GMR values for encorafenib indicate that C_max_ and AUC_last_ decreased in presence of steady-state modafinil on Day 21 by 20.2% and 23.8%, respectively (Fig. 3). However, only the decrease for AUC was statistically significant, in that the 90% confidence interval excluded 1.

**Table 2:** Summary of steady-state PK parameters of encorafenib, binimetinib, LHY746, and AR00426032 with (Day 21) and without (Day 14) modafinil coadministration (Evaluable PK population)

Parameter, Units	Encorafenib		Binimetinib		LHY746		AR00426032	
	Without modafinil (*N* = 11)	With modafinil (*N* = 11)	Without modafinil (*N* = 11)	With modafinil (*N* = 11)	Without modafinil (*N* = 11)	With modafinil (*N* = 11)	Without modafinil (*N* = 11)	With modafinil (*N* = 11)
**Cmax, ng/mL**								
*n*	11	11	11	11	11	11	11	11
GM (CV%)	3540 (55.6)	2830 (67.2)	660 (39.7)	663 (25.3)	2460 (24.7)	2510 (26.8)	25.9 (63.2)	24.7 (36.7)
Median (range)	3560 (1230–6620)	2750 (678–7150)	609 (291–1250)	686 (424–923)	2560 (1740–3860)	2560 (1490–4330)	27.5 (8.23–53.4)	23.8 (13.7–40.0)
AUClast, ng*hr/mL								
*n*	11	11	11	11	11	11	11	11
GM (CV%)	11,900 (35.8)	9100 (30.3)	2400 (38.6)	2290 (30.6)	31,100 (45.4)	33,100 (31.2)	114 (64.5)	112 (33.0)
Median (range)	11,800 (6300–19,500)	10,000 (5840–14,400)	2670 (1100–3590)	2430 (1090–3120)	33,200 (12,300–62,000)	34,000 (18,100–63,200)	127 (36.1–226)	111 (70.9–197)
**Tmax, hr**								
*n*	11	11	11	11	11	11	11	11
Median (range)	1.00 (0.88–2.03)	1.00 (0.98-3.00)	1.00 (0.88-3.00)	1.00 (0.95–2.10)	2.05 (1.00–6.00)	2.10 (1.00–6.00)	2.00 (0.88-3.00)	2.00 (0.95–2.23)
**T**_**1/2**_, **hr**								
*n*	11	11	11	11	9	10	11	11
GM (CV%)	3.48 (26.6)	3.57 (27.3)	4.62 (42.1)	4.55 (44.8)	8.67 (33.1)	8.01 (18.8)	5.60 (37.1)	5.22 (58.7)
Median (range)	3.50 (2.36–5.04)	3.80 (2.22–5.42)	5.15 (2.10–8.14)	4.47 (2.16–9.69)	8.91 (5.18–13.1)	8.08 (6.21–10.5)	5.44 (2.57–8.86)	5.75 (2.88–18.1)
**MRAUC**_**last**_								
*n*	-	-	-	-	11	11	11	11
GM (CV%)	-	-	-	-	3.31 (58.9)	4.62 (43.3)	0.0458 (65.9)	0.0468 (50.3)
Median (range)	-	-	-	-	3.89 (1.06–5.79)	5.16 (2.20–7.83)	0.0482 (0.0182–0.138)	0.0440 (0.0275–0.123)
**MRC**_**max**_								
*n*	-	-	-	-	11	11	11	11
GM (CV%)	-	-	-	-	0.883 (72.5)	1.13 (77.6)	0.0377 (53.4)	0.0358 (47.3)
Median (range)	-	-	-	-	0.917 (0.334-3.30)	1.24 (0.442-4.80)	0.0367 (0.0165–0.0926)	0.0335 (0.0192–0.0850)

MR = metabolite ratio; GM = geometric mean; N = number of patients in the treatment group; n = number of patients with non-missing values; CV% = the geometric mean CV% calculated as: sqrt (exp (variance for log transformed data)-1)*100

Four patients were excluded from the PK analysis due to significant dose interruption due to an AE, a modafinil prescription error, a missed modafinil dose on day 21, and aberrant (low) PK exposure of encorafenib on Day 14

Mean plasma concentrations of LHY746 were characterized by a steady formation phase, typically reaching peak plasma levels within 3 h postdose. Plasma concentrations of LHY746 declined with a t_1/2_ value of 8.67 h on Day 14 and 8.01 h on Day 21. Peak and total exposure to LHY746 was similar on Day 21 compared to Day 14, with GMRs of 1.02 and 1.06 for C_max_ and AUC_last_, respectively (Fig. 3). Furthermore, the metabolite to parent ratios for LHY746 reflected the above observations, with GMRs of 1.28 and 1.40 for MRC_max_ and MRAUC_last_, respectively.

#### Binimetinib and AR00426032

**Fig. 4:**
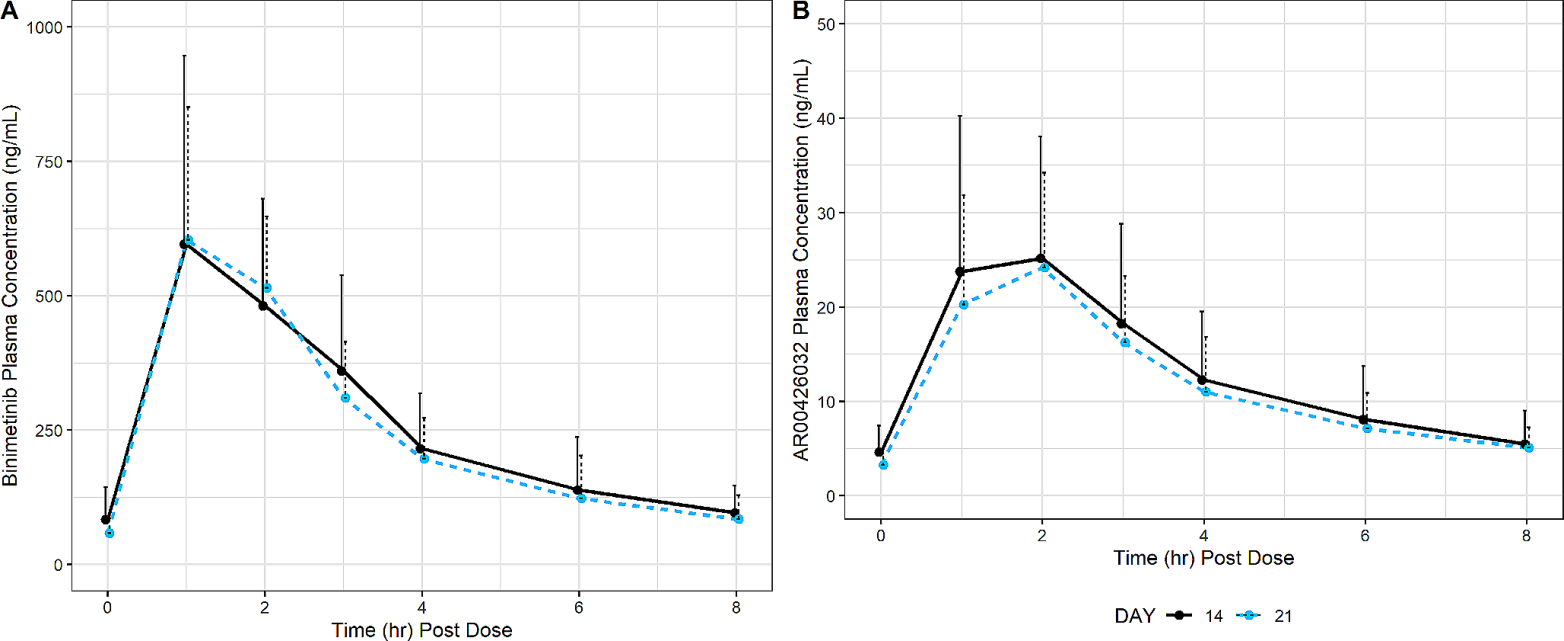
Mean (+ SD) Concentration-Time Profiles for Plasma Binimetinib and AR00426032 by Study Day. The solid lines represent the data on Day 14 and the dotted lines represent the data on Day 21. The solid dots represent the mean plasma concentrations at the time point specified. The black lines represent the upper standard deviation of the plasma concentrations at each time point. Figure 4A presents binimetinb concentrations and Fig. 4B presents AR00426032 concentrations

Following repeated daily oral administration of binimetinib for 21 days, mean plasma concentrations of binimetinib on Days 14 and 21 were characterized by a rapid absorption phase, typically reaching peak plasma levels at 1-hour postdose (Fig. 4; Table 2). The plasma concentrations declined thereafter with multiphasic disposition. Binimetinib exposures at Day 14 were consistent with historical studies [13]. Mean plasma concentrations of AR00426032 were characterized by a rapid formation phase, typically reaching peak plasma levels at 2 h postdose.

**Fig. 5:**
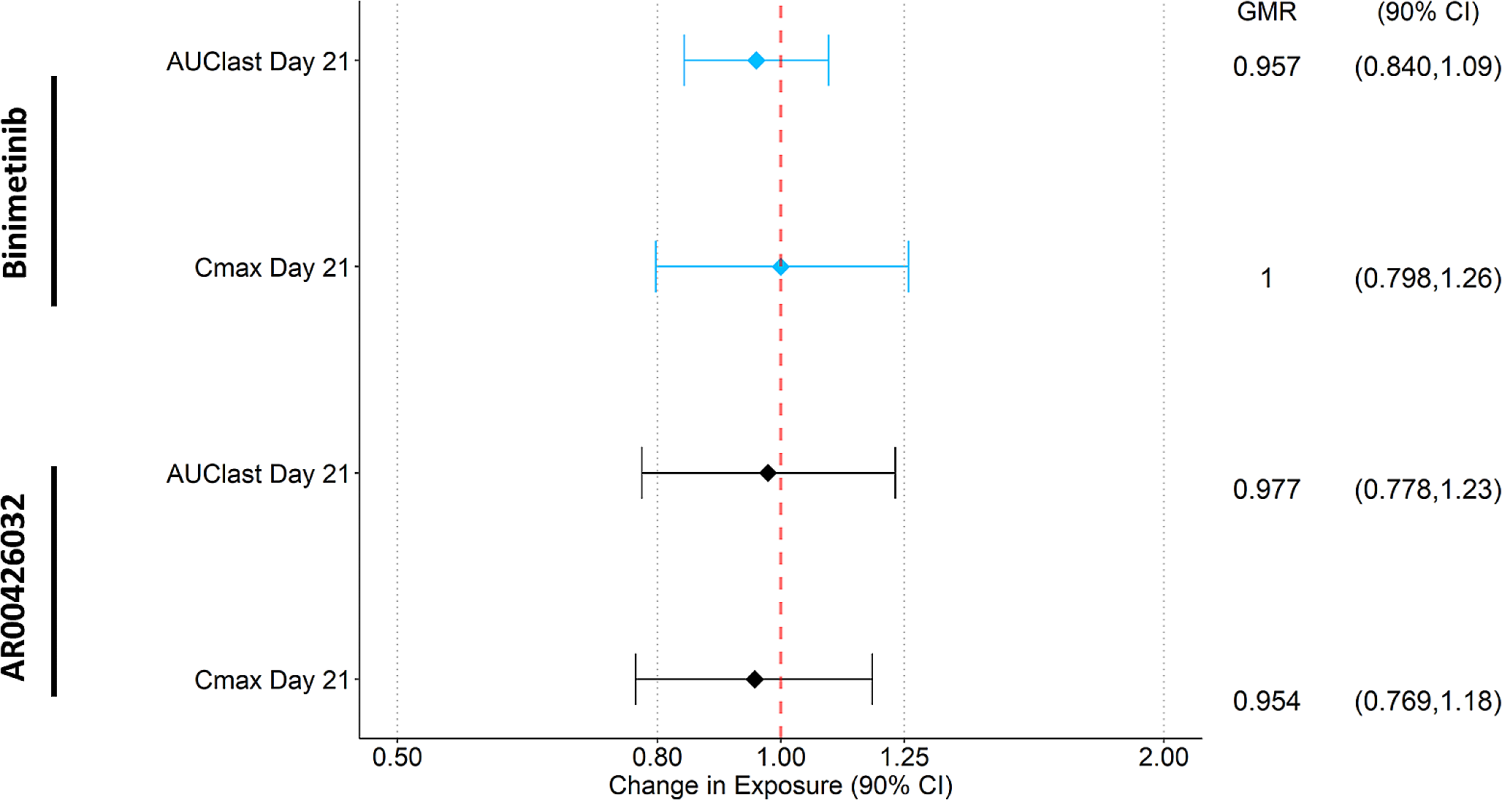
Effect of Steady-State Modafinil on Steady-state Binimetinib and AR00426032 PK Parameters. Participants were administered 450 mg QD encorafenib and 45 mg binimetinib from Day 1 to the end of the study and 400 mg QD modafinil was coadministered from Day 15-21. AUC_last_, area under the curve from 0 to the last measurable point; CI, confidence interval; C_max_, maximum observed concentration; GMR, geometric mean ratio

Peak and total exposure to binimetinib was similar on Day 21 compared to Day 14, with GMRs of 1.00 and 0.957 for C_max_ and AUC_last_, respectively (Fig. 5); this was also the case for the metabolite AR00426032, with GMRs of 0.954 and 0.977 for C_max_ and AUC_last_, respectively. Furthermore, the metabolite to parent ratios (MRC_max_ and MRAUC_last_) for AR00426032 reflected the above observations, with GMRs of 0.950 and 1.02 for MRC_max_ and MRAUC_last_, respectively.

### Safety

Of the 15 treated participants, 11 (73.3%) experienced all-causality treatment emergent adverse events (TEAEs) of any grade, 10 (66.7%) of which were considered treatment-related; 1 (6.7%) experienced all-causality TEAEs of Grade 3 or higher (decreased appetite). Four (26.7%) participants experienced TEAEs of any grade leading to study drug dose interruption. One of these four participants was discontinued from the study as they missed more than three doses of encorafenib. The other three participants did not miss more than two doses of encorafenib, allowing them to continue on the study. Induction of CYP3A usually takes 14–28 days to return to normal levels, and so missing only two doses should not impact the DDI evaluation as CYP3A should still be induced [14]. The most frequently (> 15%) reported all-causality TEAEs were diarrhoea (26.7%), nausea (26.7%), asthenia (20.0%), and headache (20.0%). No SAEs or AEs leading to study drug discontinuation/dose reduction were reported.

Overall, in adult participants with BRAF V600-mutant advanced melanoma or other solid tumors, daily doses of encorafenib in combination with binimetinib were generally safe and well-tolerated when co-administered with daily doses of CYP3A inducer modafinil. The TEAEs observed in this study were consistent with the known safety profile of the encorafenib and binimetinib combination. No new safety findings were observed.

## Discussion

As with many oral anticancer drugs, encorafenib is metabolized by CYP3A and therefore is susceptible to interactions with CYP3A inhibitors and inducers. While the effects of strong and moderate CYP3A inhibitors have been studied and recommendations for reduced starting doses of encorafenib has been included in the label, recommendations on coadministration with strong and moderate inducers, prior to this study, were based solely on in vitro data.

The results of this clinical study indicate that coadministration of multiple dose encorafenib and binimetinib with multiple doses of modafinil, considered to be a moderate CYP3A inducer at a dose of 400 mg /day, reduced the plasma exposure of steady-state encorafenib by approximately 24% when compared to encorafenib and binimetinib alone. In addition, encorafenib has a flat exposure-response relationship as observed by the lack of association between encorafenib exposure and overall response rate, time to progression free survival, and time to overall survival [5]. These results indicate that moderate inducers of CYP3A are not expected to cause a clinically significant decrease in encorafenib exposures.

In general, guidance from the EMA and FDA suggests a worst-case approach, evaluating in either healthy volunteers or patients the interaction with a strong inducer, and if significant to extrapolate the effects of moderate and weak inducers using physiologically-based pharmacokinetic (PBPK) simulations [15, 16]. In situations where clinical data for a DDI with strong CYP3A inducer rifampin were available, PBPK models were developed using the rifampin data and then used to predict the impact of weak and moderate inducers on CYP3A4 substrates [17–19]. Olaparib and osimertinib are both examples of this paradigm, where studies with rifampin resulted in a 78.5% decrease and 87% decrease in AUC, respectively, then were followed up with PBPK predictions of efavirenz (moderate CYP3A inducer) and dexamethasone (weak CYP3A inducer). Cobimetinib, a MEK inhibitor, is an example where PBPK modeling was used to predict the DDI for both strong and moderate inducers, i.e., 83% and 72% reduction in AUC, respectively. Based on simulations, the cobimetinib label contains the recommendation to avoid strong or moderate CYP3A inducers [20]. In this case, the drug approved in combination with cobimetinib is a weak CYP3A inducer (vemurafenib) and in combination was shown to result in 13% reduction in cobimetinib exposure. However, PBPK modeling to predict the effect of CYP3A induction on a drug like encorafenib that (based on in vitro data) is an inducer, inhibitor and time dependent inhibitor of CYP3A is one situation where there may be less confidence in the accuracy of PBPK predictions [21].

Another strategy to predict the effect strong induction may have on encorafenib PK, or the PK of other CYP3A substrates, but with fewer assumptions was proposed in a review by Molenaar-Kuijsten et al. [22]. Based on a review of 12 approved oral anticancer drugs primarily metabolized by CYP3A, if no clinical DDI data were available for moderate inducers, an effect of 50% compared to strong inducers could be assumed. After expanding the dataset of Molenaar-Kuijsten to include 3 additional approved oral anti-cancer drugs (fuzuloparib [23, 24], pirtobrutinib [25], and fedratinib [26]) metabolized by CYP3A, we applied this approach in the opposite direction (lack of clinical data for the effect of strong CYP3A4 inducers) given available clinical DDI data generated in this study for encorafenib (Table 3). The median ratio of the decrease for a strong inducer to a moderate inducer was 1.56 and ranged from 1.15 for cobimetinib to 2.43 for palbociclib. Therefore, the effect of a strong inducer on encorafenib exposures would be expected to be in the range of a 28–58% reduction in AUC. The upper end of this range is consistent with PBPK simulations conducted prior to the study, indicating encorafenib exposures would decrease by ∼ 58% in combination with rifampin (based on a model with some simplifying assumptions) and would also be consistent with the recommendations based on in vitro data to avoid strong inducers in combination with encorafenib [27]. This approach to managing potential coadminsitration of encorafenib with a strong inducer of CYP3A is the most conservative due to lack of more precise extrapolation methods and clincial data.

**Table 3:** CYP3A4 Oral Substrates with Oncology Indications which Included Rifampin and Efavirenz DDI Studies Reported after 2016

Compound	Effect of Strong CYP3A Inducer on AUC	Strong Inducer	Effect of Moderate CYP3A Inducer on AUC	Moderate Inducer	Strong CYP3A Inducer Effect /Moderate CYP3A Inducer Effect
Ceritinib	↓ 68.5%	Rifampin	↓ 43%	Efavirenz	1.59
Cobimetinib	↓ 83%	Rifampin	↓ 72%	Efavirenz	1.15
Olaparib	↓ 79%	Rifampin	↓ 57.3%	Efavirenz	1.38
Osimertinib	↓ 78.5%	Rifampin	↓ 42%	Efavirenz	1.87
Palbociclib	↓ 85.2%	Rifampin	↓ 35% (mean)	Modafinil: ↓ 32% Efaviranz: ↓ 38%	2.43
Sonidegib	↓ 76.6%	Rifampin	↓ 49%	Efavirenz	1.56
Fuzuloparib^a^	↓ 32%	Rifampin	↓ 22%	Efavirenz	1.45
Pirtobrutinib^a^	↓ 71%	Rifampin	↓ 49%	Efavirenz	1.45
Fedratinib^a^	↓ 81%	Rifampin	↓ 47%	Efavirenz	1.72
				Summary Statistics	
					Strong CYP3A Inducer Effect /Moderate CYP3A Inducer Effect
				Mean	1.62
				Median	1.56
				Minimum	1.15
				Maximum	2.43

^a^Added to the Molenaar-Kuijsten dataset

Evaluation of the DDI potential for oral anticancer drugs metabolized by CYP3A should take into consideration whether victim drugs have active metabolites [15, 16]. For example, induction may lead to an increase in active (potentially contributing to on-target toxicity) metabolites generated from the parent compound and therefore considerations of the exposures of both parent and metabolite can be used to estimate the effect and inform management of potential DDI. In addition, measurement of metabolite concentrations could provide useful information on the mechanism of drug interaction [15]. In the case of encorafenib, LHY746 is the primary metabolite and is generated through CYP3A-mediated metabolism, but it shows significantly less potency than encorafenib [28]. In this study, LHY746 exposure was not significantly increased following coadministration of encorafenib and modafinil when compared to encorafenib alone. Furthermore, encorafenib in combination with binimetinib was well-tolerated when administered with multiple doses of modafinil, and safety data were consistent with the known profile of the encorafenib and binimetinib combination. These findings provide further confirmation of the low liability of an interaction of encorafenib and its metabolite in combination with moderate CYP3A inducers.

Modafinil was selected as the inducer, as opposed to the other commonly used CYP3A moderate inducer efavirenz, due to due to its DDI profile allowing adequate assessment for the primary objective of the study and its safety profile, with minimal overlapping toxicity to encorafenib and binimetinib. Similar to modafinil, efavirenz is also noted to be a CYP2C19 inhibitor, however efavirenz also results in clinically significant CYP2C19 induction [29, 30]. Since CYP2C19 contributes only ∼ 16% to encorafenib oxidative metabolism, the risk of a DDI due to CYP2C19 inhibition is expected to be low. However, CYP2C19 induction could have a more significant effect on encorafenib PK. From a safety perspective, efavirenz is associated with toxicities common to binimetinib and encorafenib such as rash, gastrointestinal toxicities (including nausea and vomiting) and transaminase (alanine aminotransferase and aspartate aminotransferase) increases which could adversely affect patient compliance [31]. Lastly, neuropsychiatric effects associated with efavirenz are known to occur in up to 70% of patients, with reactions appearing as early as the first dose [32].

Although not a primary objective in this study, no change was observed for binimetinib in the presence of steady-state modafinil. Metabolism mediated by UGT1A1 is thought to contribute up to 61% of binimetinib metabolism, with no perceptible involvement of CYP3A in binimetinib metabolism. However, the mechanism for CYP3A induction is thought to potentially lead to induction of UGT1A1 also, albeit to a lesser extent [6, 7]. The lack of effect of modafinil coadministration on binimetinib PK is consistent with the understanding of binimetinib PK, with no clinically significant DDI expected from UGT1A1 inhibition or induction [8].

## Conclusion

In participants with BRAF V600-mutant unresectable or metastatic melanoma or other advanced solid tumors, encorafenib steady-state C_max_ and AUC_last_ were decreased in presence of steady-state modafinil by approximately 20% and 24%, respectively, indicating that moderate CYP3A4 inducers will not cause a clinically significant reduction in encorafenib exposures. Based on these results, no change in encorafenib dosing (alone or in combination) is recommended when coadministered with a moderate CYP3A inducer.

## Data Availability

The analyses in this paper were based on a data cutoff of 07/11/2022. Upon reasonable request and subject to review, Pfizer will provide the data that support the findings of this study. Subject to certain criteria, conditions, and exceptions, Pfizer may also provide access to the related individual de-identified participant data from Pfizer-sponsored global interventional clinical studies conducted for medicines, vaccines, and medical devices (1) for indications that have been approved in the United States and/or European Union or (2) in programs that have been terminated (that is, development for all indications has been discontinued). Pfizer will also consider requests for the protocol, data dictionary, and statistical analysis plan. See https://www.pfizer.com/science/clinical-trials/trial-data-and-results for more information. Data may be requested from Pfizer trials 24 months after study completion. The de-identified participant data will be made available to researchers whose proposals meet the research criteria and other conditions, and for which an exception does not apply, via a secure portal. To gain access, data requestors must enter into a data access agreement with Pfizer.
